# Pain Medications Used by Persons Living With Fibromyalgia: A Comparison Between the Profile of a Quebec Sample and Clinical Practice Guidelines

**DOI:** 10.1080/24740527.2023.2252037

**Published:** 2023-08-25

**Authors:** Gwenaelle De Clifford-Faugère, Hermine Lore Nguena Nguefack, Marimée Godbout-Parent, Mamadou Aliou Diallo, Line Guénette, M. Gabrielle Pagé, Manon Choinière, Sylvie Beaudoin, Aline Boulanger, Anne Marie Pinard, David Lussier, Philippe De Grandpré, Simon Deslauriers, Anaïs Lacasse

**Affiliations:** a Département des sciences de la santé, Université du Québec en Abitibi-Témiscamingue (UQAT), RouynNoranda, Québec, Canada; b Centre de recherche du Centre hospitalier universitaire de Québec–Université Laval, Quebec City, Québec, Canada; c Faculté de pharmacie, Université Laval, Quebec City, Québec, Canada; d Centre de recherche du Centre hospitalier de l’Université de Montréal (CRCHUM), Montréal, Québec, Canada; e Département d’anesthésiologie et de médecine de la douleur, Faculté de médecine, Université de Montréal, Montréal, Québec, Canada; f Patiente Partenaire, Laboratoire de recherche en épidémiologie de la douleur chronique, UQAT, RouynNoranda, Québec, Canada; g Centre d’expertise en gestion de la douleur chronique, Centre hospitalier de l’Université de Montréal, Montréal, Québec, Canada; h Département d’anesthésiologie et de soins intensifs, Université Laval, Quebec City, Québec, Canada; i Centre intégré de recherche en réadaptation et intégration sociale, Centre intégré de santé et de services sociaux (CIUSSS) de la Capitale-Nationale, Quebec City, Québec, Canada; j Institut universitaire de gériatrie de Montréal, Montréal, Québec, Canada; k Département de médecine, Faculté de médecine, Université de Montréal, Montréal, Québec, Canada; l Familiprix Chantale Gaboury & Marie-Ève Gélinas, Québec, Canada; m Groupe de médecine familiale Clinique Familiale des prairies, Québec, Canada; n VITAM–Centre de recherche en santé durable, CIUSSS de la CapitaleNationale, Quebec City, Québec, Canada

**Keywords:** fibromyalgia, pain, medications, guidelines, risk, perceived risk

## Abstract

**Background:**

Pharmacological management of fibromyalgia is complex. Chronic pain management is characterized by off-label prescribing and use, multimorbidity, and polypharmacy.

**Aims:**

This study aimed to describe pain medications use and perceived risk among people living with fibromyalgia and compare this use to evidence-based recommendations.

**Methods:**

Directive telephone interviews were conducted with 63 individuals self-reporting a diagnosis of fibromyalgia (Quebec, Canada). The questionnaire addressed specific questions about their pain and pharmacological treatments currently used for pain management (prescribed and over-the-counter). Collected data were compared to the Canadian Fibromyalgia Clinical Practice Guidelines and to evidence reports published by recognized organizations.

**Results:**

Despite a lack of robust scientific evidence to support opioids use to manage pain in fibromyalgia, 33% of our sample reported using them. Nonsteroidal anti-inflammatory drugs were used by 54.0% of participants, although this medication is not recommended due to lack of efficacy. Tramadol, which is recommended, was used by 23.8% of participants. Among the medications strongly recommended, anticonvulsants were used by 36.5%, serotonin norepinephrine reuptake inhibitor antidepressants by 55.6%, and tricyclic antidepressants by 22.2%. Cannabinoids (17.5%) and medical cannabis (34.9%) use were also reported. For all of these medication subclasses, no differences were found between participants not reporting (*n* = 35) or reporting (*n* = 28) more than one pain diagnosis (*P* < 0.05). Medication subclasses considered most at risk of adverse effects by participants were the least used.

**Conclusions:**

Results reveal discordance between evidence-based recommendations and medications use, which highlights the complexity of pharmacological treatment of fibromyalgia.

## RÉSUMÉ

**Contexte:** La prise en charge pharmacologique de la fibromyalgie est complexe. La prise en charge de la douleur chronique est caractérisée par la prescription et l’utilisation non conforme de médicaments, la multimorbidité et la polypharmacothérapie.

**Objectifs:** Cette étude visait à décrire l’utilisation de médicaments contre la douleur et le risque perçu chez les personnes atteintes de fibromyalgie, et à comparer cette utilisation aux recommandations fondées sur des données probantes.

**Méthodes:** Des entretiens téléphoniques directifs ont été menés auprès de 63 personnes ayant déclaré avoir reçu un diagnostic de fibromyalgie (Québec, Canada). Le questionnaire abordait des questions précises sur leur douleur et les traitements pharmacologiques actuellement utilisés pour la prise en charge de leur douleur (prescrits et vendus sans ordonnance). Les données recueillies ont été comparées aux Lignes directrices canadiennes sur la fibromyalgie et aux rapports de données probantes publiés par des organisations reconnues.

**Résultats:** Malgré l’absence de données probantes robustes à l’appui de l’utilisation des opioïdes pour la prise en charge la douleur chez les personnes atteintes de fibromyalgie, 33 % de notre échantillon a déclaré les utiliser. Les anti-inflammatoires nonstéroïdiens étaient pour leur part utilisés par 54,0 % des participants, bien que ce médicament ne soit pas recommandé en raison d’un manque d’efficacité. Le tramadol, recommandé, était utilisé par 23,8 % des participants. Parmi les médicaments fortement recommandés, les anticonvulsivants étaient utilisés par 36,5 % desparticipants, les antidépresseurs inhibiteurs de la recapture de la sérotonine et de la noradrénaline par 55,6 % des participants, et les antidépresseurs tricycliques par 22,2 % d’entre eux. La consommation de cannabinoïdes (17,5 %) et de cannabis médical (34,9 %) ont également été signalées. Pour toutes ces sous-classes de médicaments, aucune différence n’a été trouvée entre les participants ne signalant pas (n = 35) ou signalant (n = 28) plus d’un diagnostic de douleur (P < 0,05). Les sous-classes de médicaments considérées par les participants comme les plus à risque d’effets indésirables étaient les moins utilisées.

**Conclusions:** Les résultats révèlent une discordance entre les recommandations fondées sur des données probantes et l’utilisation de médicaments, ce qui met en évidence la complexité du traitement pharmacologique de la fibromyalgie.

## Introduction

The *International Classification of Diseases* 11th Revision of the World Health Organization in collaboration with the International Association for the Study of Pain recognizes some forms of chronic pain as a distinct health condition and not a symptom of another disease.^1^ Fibromyalgia is a chronic primary pain diagnosis under the umbrella of widespread chronic primary pain.^1^ Its diagnosis is based on a clinical evaluation,^2^ whereas the validity of biomarkers is limited.^3^ In 2018, the Analgesic, Anesthesic, and Addiction Clinical Trial Translations Innovations Opportunities and Network–American Pain Society Pain Taxonomy have proposed new diagnostic criteria for fibromyalgia.^4,5^ Some organizations or entities brought nuances to this revised definition. For example, in Quebec (Canada), the Ministry of Health “Algorithm for the Management of Fibromyalgia”^2^ underlines that (1) pain can be found on nine possible sites, (2) multisite pain plus other manifestations (i.e., fatigue or sleep problems) must have been present for at least 3 months, and (3) a full assessment has to be performed to exclude the possibility of any other differential diagnoses.

According to Queiroz,^6^ who analyzed data from 26 studies conducted on different continents, approximately 2.7% of the general population has fibromyalgia,^6^ which would represent more than 1 million and people in Canada and 8.9 million people in the United States. The prevalence of this disease is higher in women than in men, with respectively 4.2% vs. 1.4% of people affected in the general population.^6^ Fibromyalgia is more frequent in people over 50 years old, people with lower education level and socioeconomic status, and those who live in rural areas.^6^ It is also comorbid with various conditions such as headaches, depression, anxiety, chronic fatigue syndrome, and hypertension.^6^

Like chronic pain management in general, the treatment of fibromyalgia should be multimodal, including pharmacological, physical, and psychosocial approaches.^7^ According to a recent consensus published by 28 experts in fibromyalgia from various countries,^4^ treatment should be based on (1) patient education and fitness, (2) pharmacological treatment based on the individual’s needs, and (3) psychotherapy. Physical and psychosocial approaches are central to the management of fibromyalgia and its associated symptoms. For example, physical activity such as exercise, Pilates, and aquatic exercises are considered effective in reducing pain and promoting overall well-being.^5^ Physical and psychosocial approaches should usually be combined with pharmacological treatment aimed at reducing pain and comorbidities (e.g., depression, fatigue).^8^

According to the Quebec algorithm for the management of fibromyalgia and the Canadian guidelines for the diagnosis and management of fibromyalgia syndrome,^2,8^ the first-line pharmacological treatment of fibromyalgia includes antidepressants (e.g., duloxetine) and anticonvulsants (e.g., pregabalin).^4,9,10^ Opioids are not recommended, except for tramadol, which has been shown to be effective in the pharmacological treatment of fibromyalgia.^8,9^ Medical cannabis (no prescription required in Quebec) and prescribed cannabinoids (e.g., nabilone) are not mentioned in the Quebec algorithm.^2^ The Canadian guidelines^8^ suggest that a trial of prescribed cannabinoid could be initiated, in particular in patients who have severe sleep problems. Nonsteroidal anti-inflammatory drugs (NSAIDs) and acetaminophen are not recommended considering their lack of efficacy for this type of disorder.^11^

Although clinical practice guidelines are built on solid evidence from randomized control trials and meta-analyses, medication prescription and use in the clinical context can be quite different. This is especially true in the context of chronic pain management, characterized by off-label prescribing and use, multimorbidity, and polypharmacy.^12–16^ Important issues with the pharmacological treatment of fibromyalgia are difficulties patients encounter in accessing their medications and nonadherence to medications.^17–20^ According to a systematic review, the prevalence of nonadherence to pharmacological treatment in people living with chronic pain ranges from 8% to 62% across studies,^19^ which could raise questions about treatment effectiveness, tolerance, and patient preferences.^8^ Indeed, the effectiveness and tolerance of treatment vary from one individual to another.^8^ In addition, data from randomized controlled trials analyzed in evidence reports often have poor external validity to persons with chronic diseases in the community.^21–23^ Also, patients’ perceptions of the risk of their medications may influence their use, such as reluctance to use tramadol due to adverse effects and difficulties with tapering.^24,25^ It would thus seem important to assess medication use among people living with fibromyalgia and assess their perception of risk of adverse effects.

This study aimed to establish a profile of pain medications used by people living with fibromyalgia. We also compared this profile to the Canadian clinical practice guideline for the diagnosis and management of fibromyalgia syndrome and international evidence reports published by health technology assessment organizations or the Cochrane collaboration. Participants’ perceptions of the risk of adverse effects associated with each medication used was also explored.

## Materials and Methods

### Design

This descriptive study is a secondary analysis of a larger cross-sectional survey about medication used by persons living with chronic pain (all types considered; chronic pain was defined as pain that lasted or recurred for more than 3 months). **Study population**. We recruited our sample from participants of the ChrOnic Pain trEatment (COPE) Cohort,^26^ a research infrastructure resulting from the recruitment of 1935 persons ≥18 years old living with chronic pain (i.e., pain for more than 3 months^1^) and able to complete a web-based questionnaire in French. The COPE Cohort participants had originally been recruited from across all Quebec regions (*n* = 17) and were shown to be representative of random samples of Canadians with chronic pain in terms of age, employment status, level of education, pain duration, pain intensity, and most common pain locations.^26^ Compared to other chronic pain samples, persons self-identifying as women were overrepresented, probably because of the web-based recruitment strategy.^26^ The COPE Cohort participants agreed to be contacted for future research projects. From February to June 2022, 141 adults living with chronic pain from the original pool of COPE participants who were using medications to manage their pain were invited to complete a structured telephone interview about medications currently used in the context of a new project (emails were sent to COPE Cohort participants in alphabetical order until we had at least 20–30 users for main classes of medications used for pain management). Among this sample, 63 persons self-reported having received a diagnosis of fibromyalgia from a physician or a nurse practitioner. The present investigation was conducted among the subsample of 63 participants (out of 141). These 63 fibromyalgia participants were comparable to representative samples of persons living with fibromyalgia in terms of age, proportion of persons with disability related to their pain, and self-perceived general health.^27–29^

### Ethics

A letter of information and consent was attached to the initial email invitation. Informed consent was demonstrated through email feedback from participants sharing their contact information and was reconfirmed verbally at the beginning of the telephone interview. Ethics approval was obtained from the Research Ethics Board of Université du Québec en AbitibiTémiscamingue (#202001, M. Diallo).

### Patient and Public Involvement

In this study, a person with lived experience of chronic pain (SB) was involved in the development of the questionnaire and interpretation of results.

### Structured Telephone Interview

For this quantitative project, a telephone computer-assisted interview (using the SurveyMonkey platform) was chosen over a self-administered questionnaire because it can be difficult for participants to distinguish types of medications they are using. Three interviewers (trainees in the field of chronic pain pharmacoepidemiology; G.D.F., H.L.N.N., and M.G.P.) were duly trained (university courses, pretesting with patient partners, supervision by A.L.) and kept exchanging methods of doing throughout the data collection process to maximize standardization. A three-part questionnaire was used. The first part focused on the sociodemographic questions (age, sex at birth, gender, education level, employment status, self-perceived general health). The second part consisted of more specific questions about pain (duration of pain in days/months or years, diagnoses other than fibromyalgia, access to a specialized pain clinic, and one open question on the use of physical and psychosocial treatments). The third part asked about the different pharmacological treatments currently used for chronic pain management (prescribed and over-the-counter). To cover a comprehensive list of medications used by persons living with chronic pain, the Medication Quantification Scale (MSQ) 4.0 was used. This tool lists a series of analgesics, co-analgesics, and medications commonly used for sleep disorders or psychological distress concomitant with chronic pain (Supplementary Material 1). It thus allowed participants to describe pain medications currently used (yes/no) and the number of pain medications used as well as participants’ perceptions of the risk associated with each medication used. Perception of risk was defined as the potential to induce a short- or long-term adverse effects such as stomach problems, constipation, nausea, dry mouth, decreased libido, interactions with other medications, dependence, abuse, insomnia, tolerance, increased pain over time, memory, or concentration problems. The instruction was “We will go through the medications you use for your pain, psychological well-being, or sleep one by one. For each of these medications, I will ask you your perception of the extent of their adverse effect: how much these effects disturb your daily life?” The overall risk of adverse effects of each medication was assessed by the MSQ 4.0 using a 10-point numeric rating scale (0 = *no risk*, 10 = *very high risk*). The interviewers were systematic and asked for information in the following order: (1) The interviewers asked for names of all medications used by participants (prompting them to look in their cabinet or consult their list of medication provided by the pharmacy). (2) After writing down all medications listed by the participant, the interviewers asked whether they used over-the-counter medication (differences were explained and some examples were listed, because those medications were often not provided in step 1). (3) Last, interviewers specifically asked about prescribed cannabinoids (nabilone) and medical cannabis; the latter was often omitted by participants until specifically asked.

### Data Analysis

Descriptive analyses were performed with SPSS version 28 (IBM Corp., Armonk, NY, USA)^30^ to document the frequency of use for each medication subclass covered by the MQS 4.0. Frequency of use was compared between participants reporting and not reporting other chronic pain diagnoses in addition to fibromyalgia using chi-square and Fisher’s exact tests. Frequencies of some subclasses of medications were combined into larger groups (e.g., all NSAIDs together) to enable comparisons with clinical practice guidelines and evidence reports published by health technology assessment organizations or the Cochrane collaboration. The Quebec Ministry of Health “Algorithm for the Management of Fibromyalgia,”^2^ the “Canadian Guidelines for the Diagnosis and Management of Fibromyalgia Syndrome,”^8,31^ the recommendations of the Agency for Healthcare Research and Quality (AHRQ) in United States,^25,32^ and 12 Cochrane systematic reviews and meta-analyses^11,33–43^ were used to put the results obtained on the participants’ use of pain medications into perspective. Data on the perceived risk associated with each medication (0–10 scores) were not normally distributed, so medians and interquartile ranges are presented.

## Results

### Sample Characteristics

Participants’ characteristics are shown in Table 1 (*n* = 63). The mean age was 53.7 ± 10.7 years (median: 53), 96.8% were women and 100.0% had lived with pain for at least 3 years (mean duration of pain was 19.4 ± 12.9 years; median: 15). In our sample, 63.5% had a college or a university degree. Self-perceived general health varied, with 47.6% considering it to be very good or good and 52.4% considering it to be fair or poor. Almost half of the participants reported another chronic pain diagnosis in addition to fibromyalgia (44.4%), with 15.9% of people having more than two pain diagnoses. The most common additional diagnosis was osteoarthritis (20.6%). The large majority did not report having access to a specialized pain clinic (82.3%).

**Table 1.** Sample characteristics.

Variables	Mean ± SD
**Age (years)**	53.7 ± 10.7
Range	28–76
Median (IQR)	53 (14)
**Duration of pain (years)**	19.4 ± 12.9
Range	3–64
Median (IQR)	15 (15)
	*n* (%)
**Sex at birth^a,b^**	
Women	60 (96.8)
Men	2 (3.2)
**Education level**	
No high school diploma	2 (3.2)
High school diploma	13 (20.6)
Certificate or professional diploma	8 (12.7)
College education	18 (28.6)
University education	22 (34.9)
**Employment status**	
Temporary or permanent disability related to pain	22 (34.9)
Disability for a reason unrelated to pain	3 (4.8)
Work full-time	12 (19.0)
Work part-time	5 (7.9)
Retired	15 (23.8)
Other	6 (9.6)
**Self-perceived general health**	
Excellent	0
Very good	6 (9.5)
Good	24 (38.1)
Fair	26 (41.3)
Poor	7 (11.1)
**Pain diagnoses other than fibromyalgia**	
Yes^c^	28 (44.4)
No	35 (55.6)
**Access to a pain clinic^b^**	
Yes	11 (17.7)
No	51 (82.3)
**Number of pain medications**	
1	2 (3.2)
2	3 (4.8)
3	5 (7.9)
4	9 (14.3)
5 or more	44 (52.4)
10 or more	6 (9.6)
**Using physical or psychosocial treatments^b^**	
Yes	55 (88.7)
No	7 (11.1)

^a^No participants reported a gender identity different from sex at birth (female or male).

^b^Missing *n* = 1.

^c^Including conditions such as arthrosis, arthritis, cervical hernia, headaches.

IQR = interquartile range.

Polypharmacy (i.e., current use of five or more medications) was found for more than half of our sample (52.4%); excessive polypharmacy (current use of ten or more medications) was reported by 9.6% of participants. Note that the medications considered for this polypharmacy include analgesics and co-analgesics, over-the-counter medications, medical cannabis, prescribed cannabinoids, and as-needed pain medications. More than 85% of participants were using physical and/or psychosocial treatments to relieve their pain at the time of the interview (88.7%). Nonpharmacological interventions used by more than 5% of the sample included physical exercise (31.5%), meditation (25.9%), massage therapy (25.9%), heat and cold (24.1%), physiotherapy (24.1%), walking (20.4%), osteopathy (18.5%), yoga (16.7%), transcutaneous electric nerve stimulation (14.8%), chiropractic (11.1%), stretching (9.3%), resting (7.4%), psychotherapy (5.6%), and acupuncture (5.6%).

### Profile of Medications Used

Table 2 presents the proportion of users for each medication subclass included in the MQS 4.0. The five subclasses most frequently used in our sample were acetaminophen (73.0%), oral NSAIDs (60.3%), serotonin and noradrenaline reuptake inhibitor (SNRI) antidepressants (55.6%), calcium channel blocker anticonvulsants (gabapentinoids, 36.5%), and medical cannabis (34.9%). For all drug subclasses, no statistically significant differences were found between participants not reporting versus reporting other chronic pain diagnoses in addition to fibromyalgia, except for acetaminophen (respectively 85.7% vs. 57.1%; *P* = 0.011).

**Table 2.** Medications used and perceived risk for people with fibromyalgia.

	Pain medication use, *n* (%)	Stratification of pain medication use (subgroups), *n* (%)			Perceived risk (0–10)
Medication subclass	Total sample (*n* = 63)	Fibromyalgia (*n* = 35)	Fibromyalgia and other diagnoses (*n* = 28)	*P* value (comparison between subgroups)	Median (IQR)
NSAIDs—Selective cyclooxygenase 2 inhibitors; e.g., celecoxib (Celebrex)	13 (20.6)	7 (20.0)	6 (21.4)	0.889	5.0 (6.5)
NSAIDs—Salicylates; e.g., acetylsalicylic acid (aspirin)	1 (1.6)	0	1 (1.6)	—	—
Other oral NSAIDs; e.g., ibuprofen (Advil, Motrin), naproxen (Naprosyn), diclofenac, ketoprofen, meloxicam, piroxicam	24 (38.1)	15 (42.9)	9 (32.1)	0.384	3.0 (7.0)
NSAIDs—Topical agents; e.g., diclofenac sodium (Pennsaid), diclofenac diethylamine (Voltaren Emulgel)	16 (25.4)	9 (25.7)	7 (25.0)	0.948	0 (0)
Various topical agents; e.g., various compounding preparations, ketamine cream, benzocaine, lidocaine (Emla), capsaicin (Zostrix), Antiphlogistine, Myoflex, Tiger Balm	16 (25.4)	7 (20.0)	9 (32.1)	0.271	0 (0)
Acetaminophen; e.g., Tylenol	46 (73.0)	30 (85.7)	16 (57.1)	0.011	0 (1.0)
Acetaminophen in combination with an opioid; e.g., acetaminophen with codeine (Triatec30, Empracet), with tramadol (Tramacet), or with oxycodone (Percocet)	10 (15.9)	6 (17.1)	4 (14.3)	0.758	5.0 (7.25)
Short-acting opioids; e.g., codeine, fentanyl, hydromorphone, morphine, oxycodone	15 (23.8)	7 (20.0)	8 (28.6)	0.427	4.0 (8.0)
Long-acting opioids; e.g., methadone, extended-release formulations (Codeine Contin, OxyContin, Hydromorph Contin, Jurnista, MS Contin, Duragesic)	5 (7.9)	1 (2.9)	4 (14.3)	0.162	2.0 (6.0)
Opioids associated with norepinephrine reuptake inhibition; e.g., tramadol, tapentadol	15 (23.8)	7 (20.0)	8 (28.6)	0.427	2.0 (7.0)
Partial opioid receptor agonists; e.g., buprenorphine (Butrans), butorphanol	3 (4.8)	1 (2.9)	2 (7.1)	0.580	7.0 (NA)
Opioids in combination with an opioid receptor antagonist; e.g., buprenorphine/naloxone (Suboxone), oxycodone/naloxone (Targin)	2 (3.2)	0	2 (7.1)	—	5.0 (NA)
Anticonvulsants—Calcium channel blockers (gabapentinoids); e.g., pregabalin (Lyrica), gabapentin gabapentin (Neurontin)	23 (36.5)	13 (37.1)	10 (35.7)	0.907	4.0 (8.0)
Anticonvulsants—Sodium channel blockers; e.g., oxycarbazepine, lamotrigine (Lamictal), phenytoin (Dilantin), carbamazepine (Tegretol)	0	0	0	—	—
Anticonvulsants—Other; e.g., levetiracetam (Keppra), topiramate (Topamax)	4 (6.3)	2 (5.7)	2 (7.1)	0.817	7.0 (7.5)
Antidepressants—SNRIs; e.g., duloxetine (Cymbalta), venlafaxine (Effexor XR)	35 (55.6)	23 (65.7)	12 (42.9)	0.070	3.0 (7.0)
Antidepressants—Selective serotonin reuptake inhibitors; e.g., citalopram (Celexa), escitalopram, fluvoxamine (Luvox), fluoxetine (Prozac), paroxetine (Paxil), sertraline (Zoloft)	7 (11.1)	4 (11.4)	3 (10.7)	0.929	0 (5.0)
Antidepressants—Serotonin reuptake inhibitors and 5HT2 receptor antagonists; e.g., trazodone	10 (15.9)	4 (11.4)	6 (21.4)	0.318	3.5 (7.25)
Antidepressants—Specific noradrenergic and serotonergic; e.g., mirtazapine (Remeron)	1 (1.6)	0	1 (3.6)	—	—
Antidepressants—Tricyclic; e.g., amitriptyline (Elavil), nortriptyline (Aventyl), desipramine	14 (22.2)	8 (22.9)	6 (21.4)	0.892	0 (5.25)
Antidepressants—Miscellaneous; e.g., bupropion (Wellbutrin)	8 (12.7)	6 (17.1)	2 (7.1)	0.282	0.5 (2.5)
Antipsychotics; e.g., aripiprazole, chlorpromazine, clozapine, haloperidol, olanzapine (Zyprexa), quetiapine (Seroquel)	10 (15.9)	5 (14.3)	5 (17.9)	0.700	1.5 (8.0)
Barbiturates; e.g., phenobarbital, primidone	0	0	0	—	—
Benzodiazepines; e.g., lobazam, clonazepam (Rivotril), alprazolam (Xanax), bromazepam, chlordiazepoxide, diazepam (Valium), flurazepam, lorazepam (Ativan), midazolam, oxazepam	17 (27.0)	7 (20.0)	10 (35.7)	0.163	0 (5.0)
Various anxiolytics, sedatives, and hypnotics; e.g., buspirone, hydroxyzine (Atarax), promethazine, zopiclone, zolpidem tartrate	5 (7.9)	1 (2.9)	4 (14.3)	0.162	0 (6.5)
Centrally acting skeletal muscle relaxants; e.g., cyclobenzaprine (Flexeril), methocarbamol (Robax)	16 (25.4)	8 (22.9)	8 (28.6)	0.605	5.0 (2.75)
GABA (Gamma-Aminobutyric Acid)-derivative skeletal muscle relaxants; e.g., baclofen (Lioresal)	0	0	0	—	—
Miscellaneous muscle relaxants; e.g., orphenadrine (Norflex)	0	0	0	—	—
Synthetic cannabinoid (by prescription); e.g., nabilone (Cesamet)	11 (17.5)	7 (20.0)	4 (14.3)	0.553	5.0 (6.0)
Medical/therapeutic cannabis; e.g., vaporized, vaped, oral, oromucosal, topical	22 (34.9)	15 (42.9)	7 (25.0)	0.140	3.0 (7.0)
Antimigraine agents—5HT-1 receptor agonists (triptans); e.g., sumatriptan (Imitrex)	11 (17.5)	5 (14.3)	6 (21.4)	0.458	1.0 (5.0)
Antimigraine agents—Calcitonin gene–related peptide antagonists; e.g., erenumab (Aimovig)	0	0	0	—	—
Antimigraine agents—Miscellaneous; e.g., pizotifen (Sandomigran)	1 (1.6)	0	1 (3.6)	—	—
Corticosteroids—Oral; e.g., prednisone, prednisolone	1 (1.6)	0	1 (3.6)	—	—
Clonidine (antihypertensive)	2 (3.2)	0	2 (7.1)	—	7.5 (NA)
Mexiletine (anti-arrhythmic)	0	0	0	—	—

NA = not enough data to generate an IQR.

IQR = interquartile range.

The cells with dashes designate drug subclasses for which no p-value could be calculated because they were used by 0% of the study participants.

### Comparisons with Fibromyalgia Clinical Practice Guidelines and Reports

Table 3 presents the synthesis of our comparisons between pain medications used in our sample and clinical practice guidelines^2,8^ as well as evidence reports published by health technology assessment organizations^25,32^ or the Cochrane collaboration.^11,33–43^ In Canada, two drugs are authorized by Health Canada for the treatment of fibromyalgia: duloxetine (SNRI antidepressant) and pregabalin (calcium channel blocker anticonvulsant). Medication subclasses to which they belong are recommended by the Quebec “Algorithm for the Management of Fibromyalgia” and the “Canadian Guidelines for the Diagnosis and Management of Fibromyalgia Syndrome.” Among the participants, 60.3% had at least one of the two recommended drugs (31.8% of our sample were using both medication subclasses).

**Table 3.** Comparisons with guideline recommendations.

	Proportion of users in our study	Quebec Ministry of Health “Algorithm for the Management of Fibromyalgia”^2^	“Canadian Guidelines for the Diagnosis and Management of Fibromyalgia Syndrome”^8^	AHRQ—Systematic review^25,32^	Evidence-based recommendations
Medication subclass	*n* (%)	—	—	—	—
Antidepressants—Tricyclic	14 (22.2)	Strongly recommended	Recommended	Amitriptyline has no clear effect	Cochrane systematic review^42^: amitriptyline recommended but diminished pain only for a minority of patients
Antidepressants—SNRIs	35 (55.6)	Recommended	Recommended	Duloxetine and milnacipran are recommended (effect on pain quality of life). There is limited evidence on mid-term effects	Cochrane systematic review^34^: duloxetine is recommended; dose of 60 or 120 mg per day (18 studies)
Antidepressants—Selective serotonin reuptake inhibitors	7 (11.1)	Recommended (sertraline, paroxetine, and fluoxetine) if intolerant to SNRI antidepressants	Recommended	—	Cochrane systematic review^41^: no statistical or clinal improvement in pain, fatigue, and sleep. Effective for depression in this population
Anticonvulsants—Calcium channel blockers (gabapentinoids)	23 (36.5)	Recommended	Recommended starting with a low dose and then increasing (effect on pain, sleep, general condition)	Small improvement in short-term pain. Pregabalin associated with improved pain, pain response, and sleep interference (not anxiety or depression). Gabapentin has a positive effect on pain but not on quality of life	Cochrane systematic review^33^: pregabalin is safe and effective. Not enough evidence for gabapentin. For other anticonvulsants: not enough evidence for lacosamide and levetiracetam
Anticonvulsants—Sodium channel blockers	0 (0)	—	—	—	Cochrane systematic review^38^: lamotrigine is not recommended Cochrane systematic review^39^: carbamazepine could be effective for some persons, but lack of evidence
Opioids associated with norepinephrine reuptake inhibitor	15 (23.8)	Recommended for exacerbations	Recommended (pain and quality of life) for moderate to severe pain not relieved by other approaches	Lack of knowledge (one trial showing positive effect of tramadol on pain)	No Cochrane systematic review
Antipsychotics	10 (15.9)	—	—	—	Cochrane systematic review^37^: quetiapine can be considered for a time-limited period (4–12 weeks) for patients with major depression (reduce pain, sleep problems, depression and anxiety)
Medical/therapeutic cannabis	22 (34.9)	—	—	—	Cochrane systematic review^36^: no data available
Centrally acting skeletal muscle relaxants	16 (25.4)	—	Cyclobenzaprine is recommended (effect on general improvement, sleep, fatigue, depression)	Cyclobenzaprine has no clear effect	No Cochrane systematic review
Synthetic cannabinoid (by prescription)	11 (17.5)	—	May be considered, especially if sleep disorders; lack of knowledge	—	Cochrane systematic review^36^: two studies. Lack of knowledge to conclude
Acetaminophen	46 (73.0)	Recommended for other conditions such as osteoarthritis	Recommended at low dose (hepatotoxicity); lack of knowledge	—	No Cochrane systematic review
NSAIDs	34 (54.0)	Recommended for other conditions such as osteoarthritis	Recommended at low dose and for a short time, especially if osteoarthritis	Significant risk of adverse effects (serious gastrointestinal, liver dysfunction, and cardiovascular adverse effects)	Cochrane systematic review^11^: no efficacy of NSAIDs compared to placebo (six RCTs)
Various anxiolytics, sedatives, and hypnotics	5 (7.9)	—	—	—	No Cochrane systematic review
Opioids	21 (33.3)	To avoid because of the risk of misuse and overdose	Not recommended because of adverse effects. May be used in some cases according to clinical judgment; lack of knowledge	Lack of knowledge	Cochrane systematic review^35^: no RCT on oxycodone to reduce pain in fibromyalgia. Need for studies
Benzodiazepines	17 (27.0)	To avoid (risk of dependence)	—	—	Cochrane systematic review^40^: no RCT on clonazepam to reduce pain in fibromyalgia

### Perceived Risk of Pain Medications

Regarding the perceived risk of each analgesic and co-analgesic included in the MQS 4.0 (see Table 2), six received a median score ≥5 out of 10: selective cyclooxygenase 2 inhibitor NSAIDs (median score of 5), acetaminophen in combination with an opioid such as tramadol (median score of 5), partial opioid receptor agonists such as buprenorphine (median score of 7), opioids in combination with an opioid receptor antagonist such as oxycodone/naloxone (median score of 5), central-acting muscle relaxants such as cyclobenzaprine (median score of 5), and prescription cannabinoids such as nabilone (median score of 5).

## Discussion

This cross-sectional study identified pain medications used by people with fibromyalgia in clinical settings, as well as their perceived risk of adverse effects. Up to 60% of our sample used one of the two prescribed medications labeled for fibromyalgia by Health Canada at the time of the data collection. Because we had not established a medication history for each participant, it is possible that pregabalin and duloxetine had been tried in the past but were not tolerated or not effective at the prescribed dosage. Also, a great variability between participants was found on their perceptions of the risk of these two medications. Moreover, prescribed cannabinoids (e.g., nabilone) and medical cannabis use were reported despite the lack of evidence towards efficacy in this population. In addition, half of the participants reported using NSAIDs and more than a third reported using opioids despite the lack of effectiveness for fibromyalgia. Such differences between recommendations and medication use could be explained by gaps between the available evidence and what really happens in clinical practice. In fact, even if a medication is recommended by clinical practice guidelines, that does not mean that it is appropriate for all patients (e.g., insurance coverage, risk of drug interactions). Even if a medication is prescribed, it does not mean that the patient will fill the prescription or be able to tolerate it. We know that the treatment of chronic pain is characterized by off-label prescribing and use, multimorbidity, and polypharmacy,^12–16^ which adds complexity to the situation. Because we did not control for insurer status, a bias in medication consumption could be present, because some medications may not be reimbursed by the public insurer (e.g., tramadol, escitalopram).^44^ Finally, over-the-counter medications are often used without being specified by a health care professional and not all patients have access to primary care prescribers or specialized pain care. Indeed, in our sample, the large majority did not have access to a pain clinic (82.3%). In sum, a clinical perspective should be considered when interpreting and putting into practice randomized controlled trials and considered evidence from meta-analyses. This also highlights the importance of health care professional–patient partnership to promote a shared decision-making process in which the health care professional considers the patient’s preference and explains the risks associated with the use of medication.^45–47^

### Available Evidence-Based Recommendations

Differences were noted between the clinical practice guidelines for the treatment of fibromyalgia and scientific evidence. The guideline development process in Canada differs depending on the organization (conducting a computerized search of the literature, reporting the search strategy, reaching consensus using open discussion).^48^ Although the Quebec algorithm was published in 2021, the references are more than 5 years old, which could partly explain the differences between recommendations and scientific evidence. In addition, the “Canadian Guidelines for the Diagnosis and Management of Fibromyalgia Syndrome” have not been updated since 2012. It would be relevant to update those clinical practice guidelines based on recent recommendations for guideline development^49^ to support clinicians’ optimal practice.

### Medical Cannabis

More than a third of our sample used medical cannabis in various forms (34.9%), but its safety and risk are not covered in the Quebec algorithm^2^ or by the AHRQ.^25,32^ Only the “Canadian Guidelines for the Diagnosis and Management of Fibromyalgia Syndrome”^8^ state that they can be considered despite the lack of knowledge, especially for patients with sleep problems. Main adverse effects highlighted by the AHRQ were dizziness and nausea (delta-9-tetrahydrocannabinol (THC)/cannabidiol (CBD) oral spray), but a lack of knowledge remains for several outcomes (e.g., addiction/dependence, cognitive effects).^32,50^ Our result for the use of medical cannabis (34.9%) are higher than that reported by Fitzcharles et al. in a fibromyalgia sample (23.9%),^51^ but they are consistent with those of two recent Quebec studies^52,53^ showing a progressive increase in the use of prescribed cannabinoids over time and a high prevalence of cannabis use among people living with chronic pain (one out of three). For chronic pain in general, the International Association for the Study of Pain Presidential Task Force and other organizations do not recommend the use of medical cannabis and prescribed cannabinoid for chronic pain management because of the lack of knowledge on efficacy and short- and long-term safety.^50,54,55^ Further studies are needed, especially for fibromyalgia and considering that non-medical cannabis has been legal in Canada since 2018.

### NSAIDS and Acetaminophen

More than half of our study sample reported using NSAIDs (prescribed or over-the-counter) for the treatment of pain. This is higher than the figure reported by Fitzcharles et al.^51^ who noted that 34% of people with fibromyalgia used NSAIDs (participants were followed up by a rheumatologist and completed a questionnaire when attending a medical appointment). Clinical practice guidelines recommend the use of acetaminophen and NSAIDs in low doses for other chronic pain conditions associated with fibromyalgia such as osteoarthritis,^2,8^ which was the most common concomitant chronic pain diagnosis in our sample. Acetaminophen was the only type of medication for which a difference in use was found between participants with fibromyalgia only compared to participants with other pain diagnoses. Although acetaminophen was widely used, no Cochrane systematic review was found on the efficacy of acetaminophen in fibromyalgia. For NSAIDs, a Cochrane systematic review including six randomized controlled trials (RCTs) highlighted that they have no effect on pain compared to placebo in adults with fibromyalgia.^11^ Moreover, the AHRQ noted an important risk of adverse effects with NSAIDs (serious gastrointestinal, liver dysfunction, and cardiovascular adverse effects).^32^ Participants in our study reported a low perceived risk of adverse effects when using this subclass of medication. These results are important because acetaminophen and some NSAIDs such as ibuprofen or naproxen are available over the counter, which may give the impression that there is little or no risk in using them, contrary to scientific evidence. NSAIDs may not be effective in treating fibromyalgia and may act on associated symptoms (e.g., headaches) or have a placebo effect (which is not unique to NSAIDs).^56^ However, considering the adverse effects of NSAIDs, awareness among patients and health professionals regarding their prolonged use by people with fibromyalgia is needed.

### Opioids

Use of opioids for pain relief in fibromyalgia is “to be avoided” according to the Quebec algorithm^2^ and is “not recommended” by the “Canadian Guidelines for the Diagnosis and Management of Fibromyalgia Syndrome.”^8^ Adverse effects of opioid treatment for chronic pain include nausea, vomiting, constipation, dizziness, somnolence, and pruritus, in addition to serious or life-threatening adverse effects such as respiratory depression, hyperalgesia, and death.^25,57,58^ The Cochrane systematic review on oxycodone did not include any RCTs.^35^ The AHRQ only stated that there is a lack of knowledge about the use of this class of medication.^25^ Indeed, the AHRQ found only one trial on the effect of tramadol (opioids associated with an inhibitor of norepinephrine and serotonin effect) on fibromyalgia pain with a significant improvement on pain.^25^ Despite the lack of scientific evidence on opioid use, they are still prescribed and used. Indeed, in this study, more than one third of the sample reported using opioids, primarily short-acting opioids or opioids associated with a norepinephrine reuptake inhibitor. These results are comparable to the results of previous studies.^59,60^ A U.S. study also found similar results: 30% of their sample of people with fibromyalgia reported using opioids (*n* = 122).^59^ Those reporting having a prescription for opioids also reported higher baseline pain and pain inference in daily life. In a Canadian study,^60^ 33% of participants with fibromyalgia were using opioids, particularly those with more severe symptoms. The authors of this study concluded after a 2-year follow-up that it is unclear whether opioids contributed to improved health status.^60^ Also, in the present study, people living with fibromyalgia had highly variable perceived risk of adverse effects of opioids, and many adverse effects were reported during interviews. On the other hand, according to an online questionnaire administered to more than 2500 people with fibromyalgia, opioids were among the medications considered most helpful.^61^ Future research must shed light on the relevance of opioid use for fibromyalgia.

### Antidepressants

Tricyclic antidepressants (e.g., amitriptyline, nortriptyline, desipramine) were used by 22.2% of our sample. The use of tricyclic antidepressants is strongly recommended by the Quebec algorithm^2^ and recommended by the “Canadian Guidelines for the Diagnosis and Management of Fibromyalgia Syndrome.”^8^ However, the AHRQ^32^ concluded that amitriptyline does not have a clear effect of pain relief, and the authors of a Cochrane systematic review^42^ concluded that amitriptyline is recommended but effective for a minority of people living with fibromyalgia. Common adverse effects are dry mouth, cardiac rhythm abnormalities, and weight gain.^32^

SNRI antidepressants (e.g., duloxetine, venlafaxine) were widely used in our sample (55.6%). SNRI antidepressants are recommended by the Quebec algorithm^2^ and the “Canadian Guidelines for the Diagnosis and Management of Fibromyalgia Syndrome.”^8^ A Cochrane systematic review including 18 studies concluded that duloxetine is recommended at a dose of 60 or 120 mg per day.^34^ According to the AHRQ, duloxetine and milnacipran are recommended (effect on pain and quality of life), but there is limited evidence on the medium-term effects.^34^ Adverse effects with SNRI antidepressants are nausea, sedation (duloxetine, dose-related), and serotonin syndrome symptoms.^32^

Selective serotonin reuptake inhibitor antidepressants, which were not commonly used in our sample (11.1%), are effective for depression in fibromyalgia populations.^41^ However, scientific evidence from a Cochrane systematic review revealed no statistical or clinically meaningful improvement in pain, fatigue, or sleep problems.^41^

### Participants’ Perception of the Risk of Adverse Effects

Our results highlight that the medication subclasses considered most likely to have adverse effects were the least used by patients. In contrast, acetaminophen, NSAIDs, medical cannabis, SNRI antidepressants, and calcium channel blocker anticonvulsants had a lower perceived risk. Because side effects and adherence to treatment are ongoing issues for the management of fibromyalgia,^62^ considering patient perspectives on the risk of adverse effects is an important avenue to explore. Physicians, pharmacists, and nurses must focus on this issue with patients to ensure optimal management. Regarding medical cannabis, our results echo positive attitudes reported by patients^63,64^ despite published position statements and concerns regarding medical cannabis use for pain management.^54,55,65^

### Strengths and Limitations of the Study

The main limitations of our study are the small sample size, the high level of education in our sample, and the overrepresentation of women. It is possible that recruiting from an existing cohort may have resulted in a selection bias among participants with higher levels of education.^66–68^ Indeed, study participants had a higher level of education than the general population^69^ and representative samples of Canadians with fibromyalgia.^28^ Fibromyalgia is more common in women than in men,^70^ and overrepresentation of women in our sample (97%) was within the range reported in previous fibromyalgia studies (84%–97%),^61,71,72^ including a random sample of Canadians with fibromyalgia (86%).^73^ One of the strengths of this study was the use of a community sample of people living with fibromyalgia (rather than possibly more severe cases seen in rheumatology or specialized pain clinics). However, the diagnosis of fibromyalgia was self-reported in this study (potentially including milder cases not meeting criteria for a fibromyalgia diagnosis),^27^ and we did not use a fibromyalgia severity scale. Further studies are thus needed. To maintain questionnaire parsimony, only a limited number of variables were prioritized to describe our study population (e.g., no information on income, health care utilization, or types of health care professionals consulted). Analgesics and co-analgesics were also self-reported. However, a study has shown that self-reported current use of prescribed pain medications among people living with chronic pain is valid.^40^ Furthermore, we used a standardized and comprehensive tool (the MQS 4.0) to interview participants and quantify pain medication classes as well as perceived risks. Although sufficient for the description of the proportion of users of each medication subclass, our sample limited the number of respondents in terms of perceived risk (only users could provide an answer on their perceived risk), and only prevalent users were captured (those who discontinued due to adverse effects and who might have reported higher risk scores were not captured). Also, reason for the use of each medication was not reported, which may explain why use of medications that are not indicated for fibromyalgia but for other pain or comorbidities of fibromyalgia such as insomnia or depression was reported. However, no statistically significant difference was found for almost all of the drug subclasses between participants not reporting versus reporting more than one diagnosis of pain. Because our data did not include any mental health measures or specific dosage information, we cannot rule out the possibility that medications like antidepressants may have been used for depression rather than for pain. However, if this were true and the prevalence of antidepressant use for fibromyalgia-related pain was overestimated, our results would highlight an even larger disparity between guidelines and actual use of antidepressants persons living with fibromyalgia. Thus, it would be relevant to harness longitudinal administrative databases and carry out future projects of greater scope in terms of dose, frequency, and duration of medication use. Also, we did not collect data on the treating health care professionals. For the recruitment strategy, we contacted participants in alphabetical order and stopped when the sample size for the main project was reached, which could have introduced a bias.

## Conclusion

Pharmacological approaches are one aspect of multimodal treatment of fibromyalgia. Despite the limitations of the study and the inability to provide a true reflection of the medication use in all persons with fibromyalgia, our results reveal discordance between evidence-based recommendations and medication use, which highlights the complexity of pharmacological treatment of fibromyalgia and the need for greater knowledge among clinicians and patients. This is especially true in primary care practice, recognized as a setting where chronic pain management is suboptimal.^74^ Perceived risk of adverse effects influences medication use, underlining the importance of dialogue between science and society. Knowledge about pharmacological pain treatment is evolving rapidly. Thus, a possible way to reduce the gap between the results of scientific studies and clinical practice is to frequently update guidelines with active strategies to implement recommendations along with medical education. Also, from a patient empowerment and deprescription perspective, self-management strategies should be promoted.
